# Simultaneous Estimation of the Vertical Stiffness in the Knee and Hip for Healthy Human Subjects during Walking

**DOI:** 10.3390/bioengineering10020187

**Published:** 2023-02-01

**Authors:** Huan Zhao, Junyi Cao, Wei-Hsin Liao

**Affiliations:** 1 Key Laboratory of Education Ministry for Modern Design and Rotor Bearing System, Xi’an Jiaotong University, 28 Xianning West Road, Xi’an 710049, China; 2 Department of Mechanical and Automation Engineering, The Chinese University of Hong Kong, Shatin, N.T., Hong Kong 999077, China

**Keywords:** ground reaction force, knee and hip, lower limb, normal walking

## Abstract

The stiffness of lower limb joints is a critical characteristic of walking. To investigate the potential of establishing a simple and universal model to describe the characteristics related to vertical vibration during human walking, vertical stiffness is introduced at the knee and hip. A multi-mass-spring model of the human body is established in the vertical direction. In the Fourier form, results of experiments on 14 healthy adults show that the vertical displacements of joints are a function of the leg length and walking cadence, while the ground reaction force is a function of the body weight and walking cadence. The obtained universal equations of vertical displacement and ground reaction force are employed as the input parameters to the proposed multi-mass-spring model. Thus, the vertical stiffness in the knee and hip can then be estimated simultaneously by the subject’s weight, leg length, and walking cadence. The variation of vertical stiffness shows different time-varying trends in different gait phases across the entire gait cycle. Finally, the proposed model for vertical stiffness estimation is validated by the vertical oscillation of the pelvis. The average error across three gait cycles for all subjects is 20.48%, with a standard deviation of 5.44%. These results display that the vertical stiffness of knee and hip across the entire gait cycle can be directly estimated by individual parameters that are easy to measure. It provides a different view of human walking analysis and may be applied in future pathological gait recognition, bipedal robots, and lower limb exoskeletons.

## 1. Introduction

Walking is one of the most common daily activities of humans, and a large number of engineered locomotion systems are designed to emulate human walking, such as bipedal walkers [1,2], biologically inspired prosthetic limbs [3], and lower limb exoskeletons [4,5]. Research in these fields requires knowledge of the stiffness of lower limbs [6,7,8] since lower limbs act as supports and actuators in walking [9,10]. As stiffness is a multifactorial expression of the musculoskeletal system [11,12,13,14], stiffness in the lower limbs has been studied a lot [15,16,17]. There are several types of ‘stiffness’ such as leg stiffness, joint stiffness, and vertical stiffness [18,19]. Leg stiffness is the quotient of ground reaction force (GRF) and the change in leg length. The joint stiffness is the torsional stiffness, which is calculated as the quotient of the moment and joint angle for passive walking. Furthermore, the instantaneous slope of the joint’s torque-angle profile is described and defined as quasi-stiffness [20,21]. In addition, joint stiffness at the ankle, knee, and hip is typically defined as the ratio of the change in muscle moment to joint angular displacement [22,23]. Vertical stiffness is generally used to describe the linear movements that occur in the vertical direction, such as hopping and jumping [24]. It was defined as the quotient of vertical ground reaction force (VGRF) and the center of mass displacement [25].

At times, vertical stiffness and leg stiffness were used interchangeably for jumping activities, but it is actually vertical stiffness rather than leg stiffness [26]. Moreover, the relationships between the leg stiffness, vertical stiffness, and joint stiffness of the stance phase in running are compared [27]. It also illustrated that joint stiffness was associated with limb stiffness (vertical stiffness and leg stiffness). As for walking, leg stiffness is calculated as the resultant GRF in the direction of the connection between the center of pressure and hip joint center, and symmetry in bilateral leg stiffness and stiffness sharing proved useful for a more complete gait assessment in children with diplegic cerebral palsy [28].

As reviewed in [29], loading, motion, and cycle all influenced the mechanical characteristics components of walking. Walking metrics such as vertical oscillation, cadence, speed, and step length can be employed to estimate the GRF during walking by a deep learning network regression algorithm [30]. To analyze the inner relationship between stiffness and walking characteristics, some dynamic models have been established. A human gait model in two degrees of freedom was developed to calculate the time-varying stiffness of the joint, and the stiffness is found to be affected by gait pattern and cadence [31]. To realize human-like GRF patterns, an actuated dissipative spring-mass model was also proposed by introducing spring-damping units to the optimization-based minimal biped model [32]. Results illustrated that stiffness and objective weight affect the number and size of peaks in the VGRF and stance time. The vertical movement of the center of mass was related to the stabilization strategies of the double support phase and the single support phase, and the difference was also reflected in the GRF [33]. In addition, the alterations of VGRF during walking were also associated with the appearance of neurodegenerative diseases [34,35].

From the above studies, it can be summarized that the vertical characteristic is crucial for assessing the walking ability of humans. However, the vertical characteristic of joints has not been studied since the reported ‘vertical stiffness’ was at the whole-body level and the joint stiffness was focused on the moment and angle applied to them. In addition, all kinds of the mentioned ‘stiffness’ were calculated only at the stance phase and based on the measured GRF, displacement, angle, and moment, which are expensive to measure.

Therefore, the objective of this study is twofold: (i) to establish a universal gait dynamic model that can estimate both the immeasurable stiffness and measurable displacement, and (ii) to estimate the vertical stiffness of the knee and hip during walking by the individual parameters. Based on these concepts, the vertical stiffness of lower limb joints is hypothesized to be directly estimated by individual parameters like leg length, body weight, and walking cadence.

## 2. Materials and Methods

**Figure 1:**
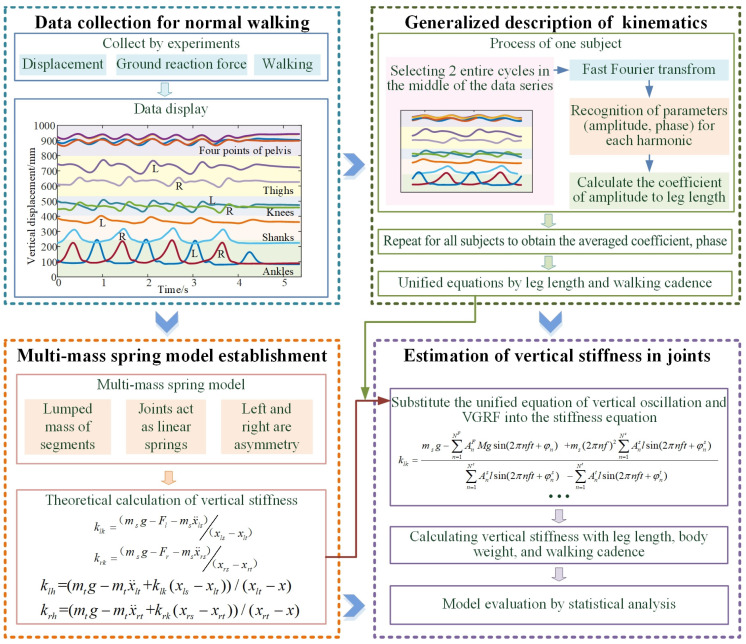
The process of the estimation of vertical stiffness by a dynamic model.

To evaluate the vertical stiffness of lower limb joints continuously and completely, a multi-mass-spring model of the lower limbs is established. Then the vertical displacements of the lower limb during walking are collected and summarized into a uniform equation. Moreover, the vertical stiffness of the hip and knee is derived. The entire process is displayed in Figure 1.

### 2.1. Subjects

This study was developed according to the Declaration of Helsinki, and all the subjects signed an approved informed consent. Lower limb displacement was measured in 14 young healthy subjects (five females and nine males; age: 25 ± 2 years old; height: 167.9 ± 10.1 cm; and body mass: 58.7 ± 10.3 kg). Subjects were free of any lower-limb musculoskeletal-related injury for at least 3 years before testing.

### 2.2. Experiments

**Figure 2:**
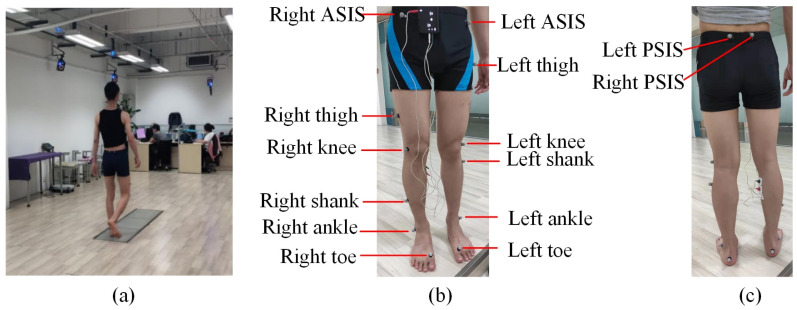
Measurement of lower limb displacement during level ground walking. (**a**) The experimental setup; (**b**) the reflective markers on the front side; and (**c**) the reflective markers on the back side.

In a gait laboratory, subjects walked at their preferred speed while wearing 16 retroreflective markers, as shown in Figure 2. The 3D trajectories are collected at 100 Hz by a 12 camera optical capture system (Vicon MX, OML, UK). The GRF was collected at 1000 Hz by three force plates (AMTI, 40060, Advanced Mechanical Technology, Inc., Watertown, MA, USA). Anthropometric parameters including height, mass, and leg length of each subject were measured and recorded. All the subjects were asked to walk barefoot at their preferred walking cadence. The distance of the walking track was about 7 m and had 3 force plates embedded in it. For all subjects, 15 trials of data were recorded for each subject.

### 2.3. Multi-Mass-Spring Model of the Lower Limbs

**Figure 3:**
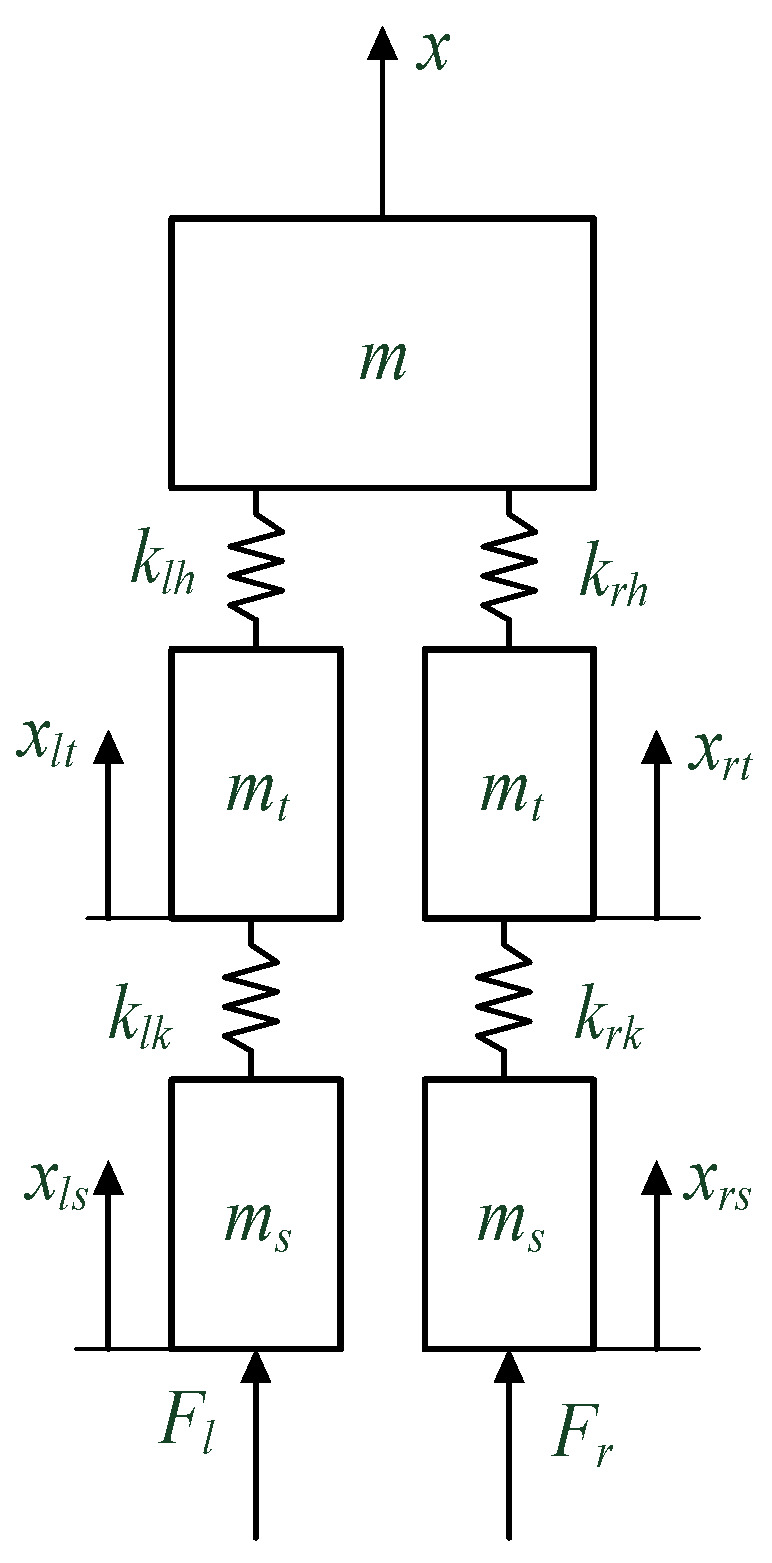
The dynamic model of the human body with ground reaction force.

A simple model that can characterize the dynamic behaviors of the lower limbs during walking is the foundation for understanding human motion. To describe the kinematics and kinetics in the vertical direction of both the left and right lower limbs, a multi-mass-spring model that includes both the knee and hip joints of the lower limbs is proposed as shown in Figure 3. The trunk and upper limbs are assumed to be concentrated mass points; moreover, the thigh and shank are both characterized as mass points.

The analytical formula can then be written as:

(1) $$\left[\begin{array}{ccccc} m_{} & 0 & 0 & 0 & 0\\ 0 & m_{t} & 0 & 0 & 0\\ 0 & 0 & m_{t} & 0 & 0\\ 0 & 0 & 0 & m_{s} & 0\\ 0 & 0 & 0 & 0 & m_{s} \end{array}\right]\left[\begin{array}{c} \ddot{x}\\ {\ddot{x}}_{lt}\\ {\ddot{x}}_{rt}\\ {\ddot{x}}_{ls}\\ {\ddot{x}}_{rs} \end{array}\right]+\left[\begin{array}{ccccc} k_{lh}+k_{rh} & -k_{lh} & -k_{rh} & 0 & 0\\ -k_{lh} & k_{lh}+k_{lk} & 0 & -k_{lk} & 0\\ -k_{rh} & 0 & k_{rh}+k_{rk} & 0 & -k_{rk}\\ 0 & -k_{lk} & 0 & k_{lk} & 0\\ 0 & 0 & -k_{rk} & 0 & k_{rk} \end{array}\right]\left[\begin{array}{c} x\\ x_{lt}\\ x_{rt}\\ x_{ls}\\ x_{rs} \end{array}\right]=\left[\begin{array}{c} mg\\ m_{t}g\\ m_{t}g\\ m_{s}g-F_{l}\\ m_{s}g-F_{r} \end{array}\right],$$

where *m* is the mass of the trunk, upper limbs, and head in total, *m_t_* and *m_s_* are the masses of the thighs and shanks, respectively, based on the relationship of the segment mass to body mass ‘*M*’ given by Leva [36], $m^{\mathrm{male}}=0.6028*M;m^{\mathrm{female}}=0.5824*M$, $m_{t}^{{}^{\mathrm{male}}}=0.1416*M;m_{t}^{{}^{\mathrm{female}}}=0.1478*M$, the foot is neglected in the model and its mass is included in the shank, $m_{s}^{\mathrm{male}}=0.057*M;m_{s}^{\mathrm{female}}=0.061*M$; *x_lt_* and *x_rt_* denote the vertical displacements of the left and right thigh, respectively; *x_ls_* and *x_rs_* refer to the vertical displacements of the left and right shanks, respectively; *F_l_* and *F_r_* are the left and right GRF in vertical, respectively; *k_lh_* and *k_rh_* indicate the vertical stiffness of left and right hip, respectively; and *k_lk_* and *k_rk_* correspond to the vertical stiffness of left and right knee, respectively.

Then the vertical stiffness of the hip and knee are derived as follows:

(2) $$\left\{ \begin{array}{l} (k_{lh}+k_{rh})x-k_{lh}x_{lt}-k_{rh}x_{rt}=mg-m\ddot{x}\\ -k_{lh}x+(k_{lh}+k_{lk})x_{lt}-k_{lk}x_{ls}=m_{t}g-m_{t}{\ddot{x}}_{lt}\\ -k_{rh}x+(k_{rh}+k_{rk})x_{rt}-k_{rk}x_{rs}=m_{t}g-m_{t}{\ddot{x}}_{rs}\\ -k_{lk}x_{lt}+k_{lk}x_{ls}=m_{s}g-F_{l}-m_{s}{\ddot{x}}_{ls}\\ -k_{rk}x_{rt}+k_{rk}x_{rs}=m_{s}g-F_{r}-m_{s}{\ddot{x}}_{rs} \end{array}\right..$$

The solution to vertical stiffness in the knee is as follows:

(3) klk=(msg−Fl−msx¨lk)(xlk−xlt)krk=(msg−Fr−msx¨rk)(xrk−xrt).

Moreover, the pelvis displacement can be derived as:

(4) Ax2+Bx+C=0A=−2mtg+2mtx¨lt−klk(xls−xlt)−mtg+mtx¨rt−krk(xrs−xrt)B=mtgxrt−mtx¨ltxrt+klk(xls−xlt)xrt+mtgxlt−mtx¨rtxlt +krk(xrs−xrt)xlt+mtgxlt−mtx¨ltxlt+klkxlsxlt−klkxlt2 +mtgxrt−mtx¨rtxrt+krkxrsxrt−krkxrt2+(mtg−mtx¨lt)(xlt+xrt)C=−2xltxrtmtg+mtxltxrt(x¨lt+x¨rt)−klkxltxrtxls+klkxlt2xrt −krkxltxrtxrs+klkxltxrt2−xltxrt(mtg−mtx¨lt)x=−B±B2−4AC2A.

The stiffness of the hip can be described as:

(5) $$\begin{array}{l} k_{lh}=(m_{t}g-m_{t}{\ddot{x}}_{lt}+k_{lk}(x_{ls}-x_{lt}))/(x_{lt}-x)\\ k_{rh}=(m_{t}g-m_{t}{\ddot{x}}_{rt}+k_{rk}(x_{rs}-x_{rt}))/(x_{rt}-x) \end{array},$$

therefore, the outputs of the model are hip stiffness, knee stiffness, and the vertical displacement of the pelvis, and they can be calculated from the inputs such as the ground reaction force, mass, and vertical displacement of the thighs and shanks. As for the vertical displacement of both left and right thighs and shanks, they can be represented with anthropometric parameters as conducted in the following section.

### 2.4. Generalized Description of Kinematics and VGRF

**Figure 4:**
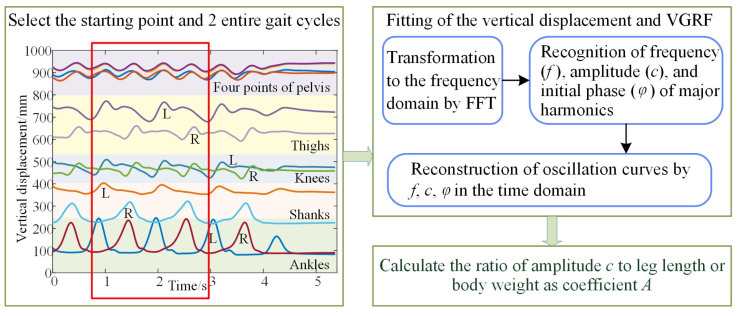
The generalizing process of the lower limb displacement description.

The collected gait signals in Section 2.2 are analyzed with the process shown in Figure 4.

**Figure 5:**
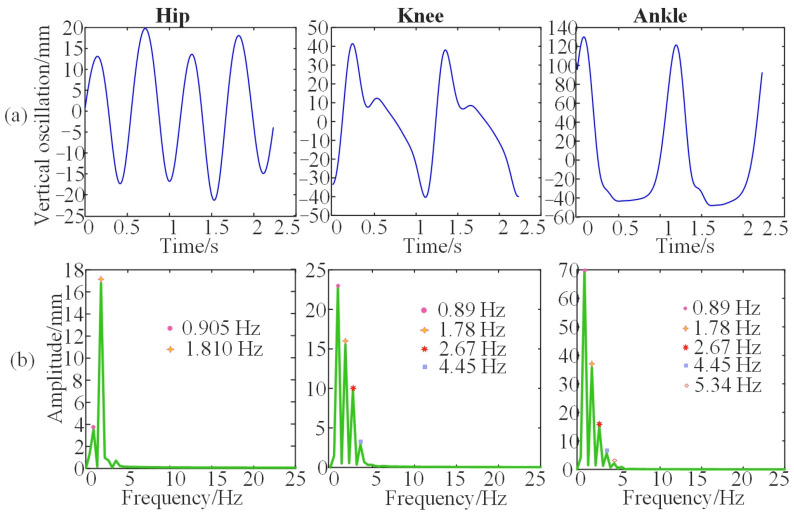
The vertical displacement and the spectrum of the joints for one subject. (**a**) The vertical oscillations of hip, knee, and ankle. (**b**) The spectrums of vertical oscillations of hip, knee, and ankle.

Because the collected gait signals begin and end with standing, the initial and final effects should be eliminated by selecting data points from the median segment. Firstly, the starting point of stable walking and 2 entire gait cycles are selected for analysis. Then the fast Fourier transformation (FFT) is used to transform the signal into the frequency domain since gait is quasiperiodic. The frequency and amplitude of major harmonics are then recognized from the frequency domain, as displayed in Figure 5.

It can be observed in Figure 5b that the vertical oscillation of the hip is mainly accumulated at the first two harmonics, while the vertical oscillation of knee is mainly at the first three harmonics, and the vertical oscillation of ankle is composed mainly of the first four harmonics.. Therefore, the vertical displacement of the hip, knee, and ankle can be represented by the two, three, and four harmonics, respectively. The Fourier series is considered to fit the oscillation trajectory of the lower limb as follows:

(6) $$S_{N}x=\frac{a_{0}}{2}+\sum_{n=1}^{N}\left(a_{n}\cos2\pi nx+b_{n}\sin2\pi nx\right).$$

The sine component and the cosine component of the same frequency can be synthesized into a sine component represented as:

(7) $$S_{N}x=\frac{a_{0}}{2}+\sum_{n=1}^{N}c_{n}\sin(2\pi nx+\varphi_{n}),$$

where $c_{n}=\sqrt{a_{n}{}^{2}+b_{n}{}^{2}}$ refers to the amplitude of each harmonic and φn=arctan(anbn) is the initial phase of the harmonic component in each order; *N* is the number of the harmonic order. The amplitude is assumed to be proportional to the leg length; therefore, the amplitude of each harmonic in the series is then divided by the leg length of the subject, and thus the ratio of amplitude to leg length is obtained. Then the mean of the ratio and the initial phase of all the subjects are calculated for a general description of lower limb displacements. Finally, the change in vertical displacement can then be derived as:

(8) $$y=\sum_{n=1}^{N}A_{n}l\sin(2\pi nft+\varphi_{n}),$$

where $A_{n}$ is the coefficient of each harmonic, *l* is the leg length of the subject, *f* refers to the real walking cadence, and it is the number of strides in one second; thus, it can also be calculated by the gait cycle time *T* since f=1T.

The theoretical displacement of one limb can also be derived from the contralateral limb since human walking has the characteristics of symmetry both in space and time. The locomotion of one limb lags a half-gait cycle compared to the contralateral limb. Thus, if a half-gait cycle is introduced to Equation (8), which means *t* in Equation (8) becomes (t−T2), then the oscillation of the contralateral lower limb joints can be expressed as:

(9) $$y_{r}=\sum A_{i}l\sin(2\pi ift+\varphi_{i}+(i-1)\pi )+A_{j}l\sin(2\pi jft+\varphi_{j})\;\;\;i=1,3,\ldots ;j=2,4,\ldots ,$$

where *i* represents the order of the odd harmonics, and *j* refers to the order of the even harmonics.

The measured VGRF is also a quasiperiodic signal, as displayed in Figure 4. Similar to the dealing process for kinematic signals, the VGRF can also be represented as:

(10) $$F=\sum_{n=1}^{N}A_{n}Mg\sin(2\pi nft+\varphi_{n}),$$

(11) $$F_{r}=\sum A_{i}Mg\sin(2\pi ift+\varphi_{i}+(i-1)\pi )+A_{j}Mg\sin(2\pi jft+\varphi_{j})i=1,3,\ldots ;j=2,4,\ldots ,$$

where *F* refers to the VGRF of one foot and *F_r_* is the VGRF of the other foot, *M* is the mass of the body, and $M=m+2m_{t}+2m_{s}$.

**Figure 6:**
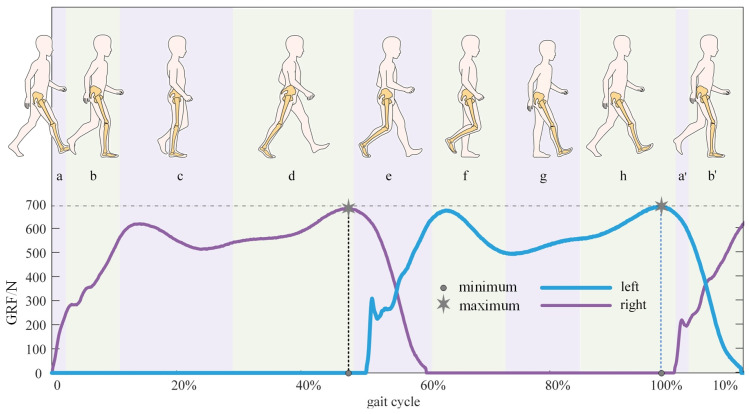
Gait cycles and corresponding ground reaction force. (**a**) Initial contact when heel strike, and it accounts for 2% gait cycle; (**b**) loading response that means foot flatting, and it accounts for 10% gait cycle; (**c**) midstance, and it accounts for 17% gait cycle; (**d**) terminal stance when heeling off, and it accounts for 19% gait cycle; (**e**) pre swing means toe-off, and it accounts for 12% gait cycle; (**f**) initial swing, and it accounts for 13% gait cycle; (**g**) mid swing, and it accounts for 12% gait cycle; and (**h**) terminal swing, and it accounts for 13% gait cycle; (**a’**,**b’**) are phases in the next gait cycle and their determination are the same as (**a**,**b**) respectively.

Walking is commonly studied as a repetitively periodic activity using the “gait cycle” [37]. The gait cycle is defined as the duration from the heel strike to the next heel strike of the same limb. It can also be subdivided into the stance phase (accounts for 60% of the gait cycle) and the swing phase (which accounts for 40% of the gait cycle). Moreover, the stance phase and the swing phase can be further subdivided, respectively. These phases can be determined based on the change in VGRF. The details of each gait phase and its corresponding VGRF are shown in Figure 6.

### 2.5. Estimation of Vertical Stiffness in Joints

The vertical oscillation of both the left and right thighs and shanks, as well as the VGRF, are represented in Equations (8)–(11) by individual parameters in a universal form. Therefore, by substituting Equations (8)–(11) into Equations (3)–(5), the vertical stiffness of the hip and knee can then be theoretically derived. Here, an equation of vertical stiffness for the left knee is displayed as:

(12) $$k_{lk}=\frac{m_{s}g-\sum_{n=1}^{N^{F}}A_{n}^{F}Mg\sin(2\pi nft+\varphi_{n})+m_{s}{\left(2\pi nf\right)}^{2}\sum_{n=1}^{N^{s}}A_{n}^{s}l\sin(2\pi nft+\varphi_{n}^{s})}{\sum_{n=1}^{N^{s}}A_{n}^{s}l\sin(2\pi nft+\varphi_{n}^{s})-\sum_{n=1}^{N^{t}}A_{n}^{t}l\sin(2\pi nft+\varphi_{n}^{t})},$$

where the superscript *F* indicates VGRF, *s* refers to the shank, and *t* corresponds to the thigh. Other theoretical equations, like the vertical stiffness of the right knee and hip, are obtained with the same process as Equation (12).

### 2.6. Statistical Analysis

The distributions of individual parameters such as body weight and height are near normal since they were tested using the Shapiro–Wilk test (*p* > 0.05) [38]. To obtain more accurate descriptions, the coefficient, initial phase, and walking cadence are averaged across the two selected gait cycles for all the subjects. Moreover, the average value and standard deviation of the model errors from all the subjects were calculated to evaluate the dynamic model. All calculations and statistical analyses in this study were carried out using MATLAB (9.6.0.1072779 (R2019a)).

## 3. Results

### 3.1. The Empirical Parameters of Unified Representation

**Figure 7:**
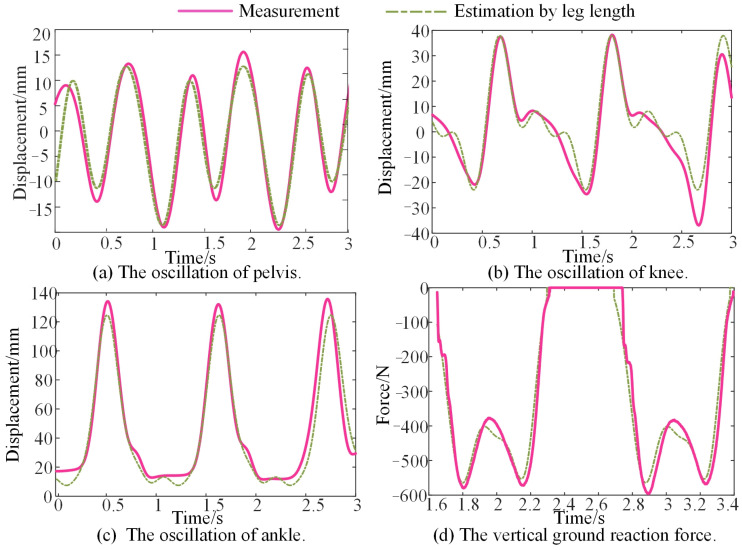
Comparison of vertical displacement between the measurement and the estimation by the individual parameters.

As obtained from Section 2.4, all the vertical oscillations of lower limb joints and segments can be obtained with amplitude coefficients and initial phases as represented in Equations (8) and (9). Furthermore, Equations (10) and (11) represent the VGRF with amplitude coefficients, initial phases, walking cadence, and body weight. Their average value across all the subjects is obtained as illustrated in Section 2.5, and they are displayed in Table 1. The vertical displacement of the lower limb can be expressed directly with leg length and walking cadence using these parameters. Moreover, the estimated vertical oscillations were compared to the measured data, as shown in Figure 7. It can be seen that the unified equation with the empirical parameters obtained in Table 1 fits the measured oscillation of the lower limbs well.

This unification of the quantitative description of human lower limb oscillation during overground walking helps to establish a general representation of the dynamic characteristics such as stiffness.

### 3.2. The Vertical Stiffness of the Knee

**Figure 8:**
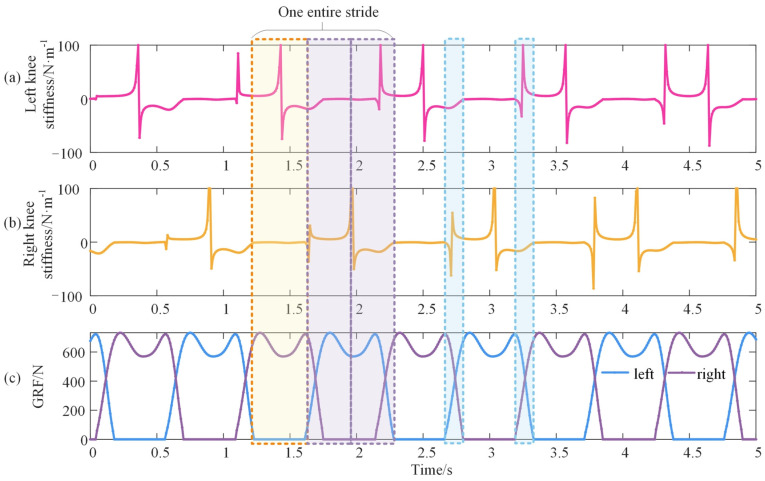
Vertical stiffness of the knee during walking. (**a**,**b**) denote the left and right knees, respectively, and (**c**) is the corresponding vertical GRF. The yellow rectangle shows the loading response phase to the terminal stance phase of the right leg and the mid-swing phase of the left leg. The purple rectangle represents the terminal stance phase to the loading response phase and is equally separated by the mid-swing phase of the right leg as well as the stance phase of the left leg. The blue rectangles are the double support phase.

The vertical displacements of the thigh and shank can be represented by the leg length and walking cadence, as illustrated in Equation (12). Figure 8a shows the obtained vertical stiffness of the left knee across several gait cycles after substituting the empirical coefficients and initial phase shown in Table 1 into Equation (12) and then calculating it with MATLAB 2019 a. With the same process, the vertical stiffness of the right knee is calculated and displayed in Figure 8b. In addition, the corresponding ground reaction force is shown in Figure 8c. It can be observed that the vertical stiffness in the knee experienced three changing stages in one stride cycle.

As shown in Figure 8, the vertical stiffness of the knee fluctuated around zero during the first 40% of the gait cycle, from the loading response phase to the terminal stance phase. Moreover, this duration equals the swing duration of the contralateral leg. At the terminal stance phase, the vertical stiffness of the knee appears as the discontinuity point of the first kind, and then it maintains a wide ‘U’ shape until the mid-swing phase with the duration of 30% of the gait cycle. There is also a discontinuity point of the first kind at the mid-swing phase, and a curve similar to a sinusoid is produced from the mid-swing phase to the loading phase with a duration of 30% of the gait cycle. The duration of the ‘U’ shape and the sinusoid stiffness curve is the exact stance duration of the contralateral leg. Furthermore, the changing tendencies of the two double support phases differ. When the limb is preparing to swing, there is a discontinuity, and when the limb is preparing for stance, the stiffness variation is continuous. Additionally, the vertical stiffness of the right knee is delaying or ahead of the left knee by half of the gait cycle.

### 3.3. The Vertical Stiffness of the Hip

**Figure 9:**
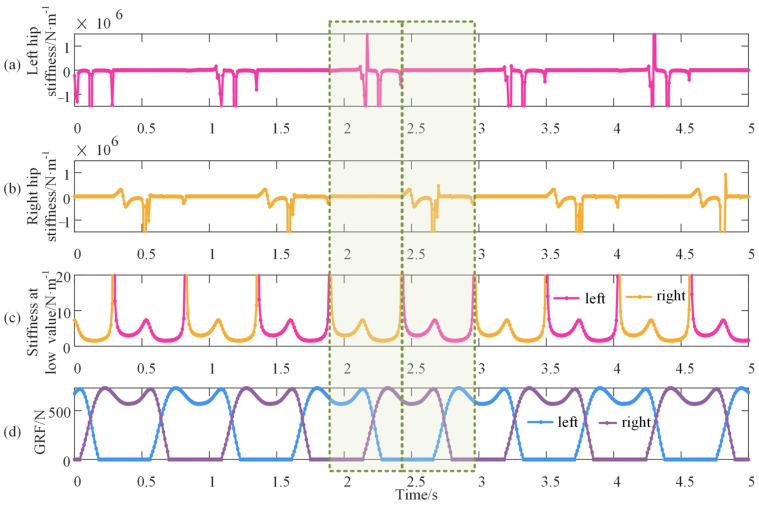
Vertical stiffness of the hip during walking. (**a**,**b**) denote the left and right hip, respectively, and it is evident that the fluctuation is rather high with a magnitude of 10^6^; (**c**) shows the hip stiffness at the section of low value; and (**d**) is the vertical GRF of the double lower limbs. The green rectangles stand for half of the gait cycle.

The empirical coefficients and initial phases of thighs and shanks shown in Table 1 are substituted into Equations (8)–(11) and Equation (4), and these equations are then substituted into Equation (5) to calculate the vertical stiffness of the hip. The obtained vertical stiffness of the left and right hips is shown in Figure 9a,b, respectively. The maximum value of hip stiffness reaches approximately 1 × 10^6^ N/m, and its fluctuation accounts for half of the gait cycle. The stiffness in another half cycle is approaching zero, which seems unchanged. To study the unchanged section, the highly fluctuating section is hidden, as shown in Figure 9c. The theoretical GRF of both lower limbs is shown in Figure 9d to recognize the corresponding gait phase of the two sections.

The vertical stiffness of the hip is extremely high when its corresponding leg goes from the mid-stance phase to the mid-swing phase. From the mid-swing phase to the mid-stance phase, the vertical stiffness of the hip is rather small at about 5 N/m but with a regular shape like ‘w’. There is a discontinuity of the first kind at the mid-stance phase and the mid-swing phase. Furthermore, the vertical stiffness between the right and left hips, like the knee, has a time delay for half of the gait cycle. During walking, the vertical stiffness of the knee and hip varies with the gait phase (time).

### 3.4. Validation of the Model

**Figure 10:**
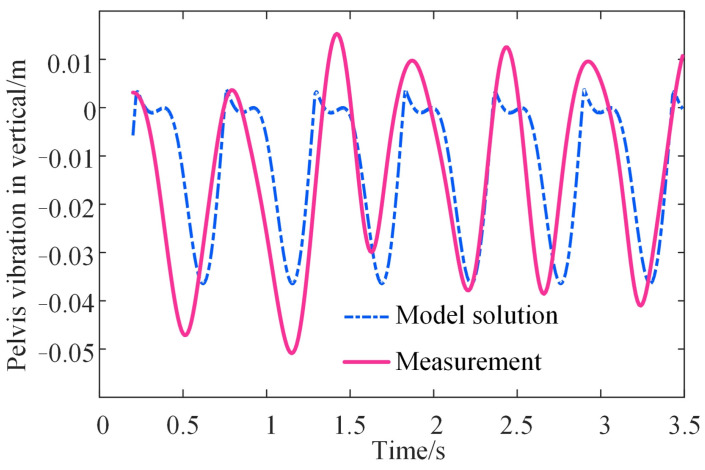
The pelvis trajectory calculated by the model and real measurement.

It is reasonable to validate the model by evaluating the pelvis displacement estimated by the model because the vertical stiffness has been difficult, if not impossible, to measure during walking until now. Errors between the model solution and the measured displacement of the pelvis are calculated. The measured displacement was collected in the experiment in Section 2.2. It contains three strides, and each stride has differences in oscillation. Therefore, the variable of time ‘*t*’ in Equation (8) is set to 3.5 s in order to include three strides. With the empirical coefficients and phases in Table 1, the vertical displacement of the thigh and shank is expressed and substituted into Equation (4). The obtained vertical displacement of the pelvis for one subject is then compared to the measured displacement as presented in Figure 10.

It can be observed that the model solution of the pelvis displacement is approximately consistent with the measured displacement. This proved that the proposed model could characterize walking characteristics such as vertical stiffness and pelvis oscillation by leg length, body weight, and walking cadence. Moreover, to illustrate the universality and stability of the identification process, the model error is calculated from the solved pelvis displacement and the measured pelvis displacement as follows:

(13) $$E=\frac{1}{t*F_{s}}\sum_{nt=1}^{t*F_{S}}\frac{x_{nt}^{\mathrm{measure}}-x_{nt}^{\mathrm{solutiom}}}{x_{nt}^{\mathrm{measure}}},$$

where *t* equals 3.5 s as mentioned before, *F_s_* equals the sampling frequency of the motion capture system, which is 100, and subscript *nt* refers to the number of time points. The errors for all the subjects are shown in Table 2. For different individuals, the errors range from 11.94 to 29.14%, and the mean error is 20.48% while the standard deviation is 5.44%.

## 4. Discussion

The primary aim of this study is to estimate the vertical stiffness of the knee and hip using individual parameters that are easy to measure during walking. To achieve this aim, a multi-mass-spring model was established. Furthermore, the Fourier series was used to fit the vertical displacements of lower limb segments and VGRF required in the established model with individual parameters such as leg length and walking cadence. According to the established model, the vertical stiffness of the knee and hip was estimated by leg length, body weight, and walking cadence across the entire gait cycle. Furthermore, the established lower limb model was validated by its solution of pelvic displacement and real measurement.

Firstly, our results implied that the established multi-spring model is effective at characterizing walking characteristics. There were different dynamic models for stiffness calculation, as shown in Table 3. A typical human gait model using a nonlinear angular spring and dash pot at each point was established to find the optimum joint stiffness of the hip and ankle in the stance phase [31]. It also found that stiffness variation was affected by gait pattern and cadence. An actuated dissipative model combining the optimization-based minimal biped model and the spring-loaded inverted-pendulum model was established for the stance phase, and 2 × 104 N/m (5 × 10^3^ to 1 × 10^5^ N/m) of the leg stiffness achieved the closest GRF profile [32]. This supported our finding that the vertical stiffness of the hip in the stance phase is sometimes varied at a high value level, as displayed in Figure 9. The quasi-stiffness of the knee and ankle was predicted using statistical models based on subject weight and height [18,20]. They provided the foundation for the idea that immeasurable characteristics can be predicted by measurable parameters. A point mass with two massless springs was also established as a dynamic model to calculate the leg stiffness in the stance phase [39] and to predict the trajectory of the center of mass. Compared to these dynamic models for stiffness estimation in the stance phase, the multi-mass spring model established in this study can estimate stiffness across the entire gait cycle, and its solution of pelvis displacement has been validated.

Aside from the ability of the proposed model in our study to be consistent across the entire gait cycle, the vertical stiffness of joints in our study was a different concept from traditional joint stiffness. Traditionally, the stiffness of the knee and hip was calculated as the quotient of the moment and joint angle change in the sagittal plane, and the moment was calculated by the trajectory data and the GRF [40]. This joint stiffness illustrated the relationship between the angle and the corresponding moment applied to the joints during walking. While the vertical stiffness of the joints investigated in this study shows a link between vertical oscillation of lower limb segments and VGRF.

During the model solution, the vertical oscillations of lower limb segments and VGR were utilized. The vertical oscillation of lower limb segments was fitted by the Fourier series with leg length and walking cadence, while the VGRF was represented with body weight and walking cadence. The amplitude coefficient and initial phases shown in Table 1 contributed to a universal and mathematical expression. These findings were supported by previous findings. Fourier series, for example, had been used to characterize the pelvic trajectory [41].

Since the vertical stiffness of the knee and hip was obtained solely by individual parameters such as body weight, leg length, and walking cadence, which are all easy and cheap to measure, it implies that VGRF, body weight, and vertical oscillation of body segments have inherent relationships. This is similar to the previous research. The body weight influenced the GRF, and the vertical displacement of the body for a given individual was determined by the effective leg length [32]. Furthermore, it was demonstrated that the VGRF estimated the vertical displacement of the body mass [42].

Moreover, when compared to previous studies, our study illustrates the time-varying process of vertical stiffness corresponding to the gait phase across the entire gait cycle. In vertical, knee stiffness is near zero in the midstance and high in the terminal stance and initial swing. These findings are consistent with previous research, which found that the knee stiffness determined by the slope of the knee moment-angle curve is approximately zero at the start of the stance and increases in the late stance [21]. In addition, it is worthy to note that the vertical stiffness of the knee across the entire cycle is varied in the same order of magnitude while being different for the hip.

The contributions of this study are as follows: (1) the uniform equation to depict the vertical oscillations and VGRF of different people is obtained with individual walking cadences as well as leg length and body weight, respectively; (2) the multi-mass-spring model is established to identify the vertical stiffness of hip and knee simultaneously, and this stiffness can be represented by the body weight, leg length, and walking cadences; and (3) the obtained vertical stiffness is validated by the comparison between the estimated displacement and the measured displacement of the pelvis.

There are also some limitations that need to be considered. The main limitation is the size of the subject. Fourteen subjects walked at their preferred speed, obtaining a homogeneous sample. The analyses could be generalized only to the range of age, height, and walking cadence that the statistical significance supports. Similar estimations could be carried out for other groups, such as older adults and children. Another limitation is that several simplifications were employed. Both the mass and length of the left and right lower limbs were regarded as the same, and the ankle and foot were ignored. A more sophisticated model could be considered to take the asymmetrical factors and eliminated terms into account.

## 5. Conclusions

In summary, the vertical stiffness of the knee and hip can be simultaneously estimated by a multi-mass-spring model. It has been found that the vertical oscillations of lower limb segments were universally expressed by walking cadence and leg length, while vertical ground reaction force was represented by walking cadence and body weight. Moreover, the vertical stiffness of the knee and hip were finally estimated by the walking cadence, leg length, and body weight. The variation of the estimated vertical stiffness across the entire gait cycle displayed different trends toward different gait phases. Additionally, the proposed model was validated efficiently by the estimated vertical oscillation of the pelvis across three gait cycles for the 14 different subjects. The remarkable results obtained in this study represent a different view for future studies on human walking analysis. In the near future, more sophisticated models that consider ankle and damping will be constructed and extended to more human groups.

## Figures and Tables

**Table 1:** The parameters of the vertical displacement of the lower limbs.

Parameters	Pelvis	Thigh	Knee	Shank	Ankle	VGRF
*A*_1_	0.007	0.014	0.019	0.022	0.073	0.58
*A*_2_	0.018	0.014	0.017	0.019	0.040	0.08
*A*_3_	0	0.009	0.011	0.016	0.018	0.22
*A*_4_	0	0	0	0.006	0.006	0.03
*A*_5_	0	0	0	0	0	0.07
*A*_6_	0	0	0	0	0	0.03
*φ*_1_	−0.93	−0.59	−0.59	1.08	1.08	2.47
*φ*_2_	−0.49	−1.04	−1.04	0.61	0.61	3.18
*φ*_3_	0	−2.14	−2.14	0.10	0.10	0.98
*φ*_4_	0	0	0	0	0	3.13
*φ*_5_	0	0	0	0	0	−0.42
*φ*_6_	0	0	0	0	0	1.15

**Table 2:** The error of the estimated pelvis displacement by model.

No	1	2	3	4	5	6	7	8	9	10	11	12	13	14
Errors (%)	29.14	18.87	11.94	23.22	27.95	24.00	16.45	23.49	15.47	27.95	16.48	17.76	18.34	15.62

**Table 3:** The comparison between the typical and proposed dynamic models for stiffness calculation.

Models	Components	Aim Stiffness	Gait Phase	Input Parameters
Two-link conceptual model [31]	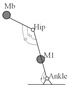	Joint stiffness of hip and ankle	Stance	Joint angle and moment
Spring-mass walking model [32]	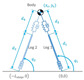	Leg stiffness	Stance	Leg length and position
Statistic model [18,20]	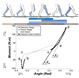	Quasi-stiffness of knee and hip separately	Stance	Body weight, height, and walking speed
Mass-spring model [39]	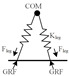	Leg stiffness	Stance	Angle and leg length change
Multi-mass spring model Proposed in this paper	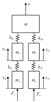	Vertical stiffness of knee and hip simultaneous	Entire gait cycle	Body weight, leg length, and walking cadence

## Data Availability

The data presented in this study are available on request from the corresponding author.
